# Liposome-Encapsulated Carfilzomib as a Radiosensitizer in Solid Tumors

**DOI:** 10.1021/acs.molpharmaceut.5c01534

**Published:** 2026-02-17

**Authors:** Matthew Molinaro, Pranay Saha, David Skrodzki, Mitchell Machtay, Dipanjan Pan

**Affiliations:** † Department of Engineering Science and Mechanics, 8082 Pennsylvania State University, University Park, Pennsylvania 16802, United States; ‡ Department of Nuclear Engineering, Pennsylvania State University, University Park, Pennsylvania 16802, United States; § Department of Materials Science and Engineering, Pennsylvania State University, University Park, Pennsylvania 16802, United States; ∥ Department of Radiation Oncology, Pennsylvania State University College of Medicine, Hershey, Pennsylvania 17022, Unites States; ⊥ Department of Biomedical Engineering, Pennsylvania State University, University Park, Pennsylvania 16802, Unites States; # Huck Institute of the Life Sciences, University Park, Pennsylvania 16802, United States; ¶ Department of Chemistry, Pennsylvania State University, University Park, Pennsylvania 16802, Unites States

**Keywords:** carfilzomib, chemoradiotherapy, abscopal effect, liposome, immunotherapy

## Abstract

Chemoradiotherapy is a common treatment option for many cancers. Carfilzomib (CFZ) is an effective chemotherapeutic drug with a multitude of cellular effects. However, CFZ has yet to be studied in the context of chemoradiotherapy. To study the application of CFZ in chemoradiotherapy, we synthesized CFZ-loaded liposomes. We report a novel liposomal formulation of the proteasome inhibitor CFZ designed to enhance tumor radiosensitivity while improving drug solubility and tolerability. CFZ-loaded PEGylated liposomes were synthesized via thin-film hydration and probe sonication, achieving an average diameter of ∼127 nm and an encapsulation efficiency of 64%. In murine 4T1 breast carcinoma cells, CFZ treatment prior to irradiation significantly reduced clonogenic survival (dose enhancement factor = 1.26) and increased γ-H2AX foci retention, indicating impaired DNA double-strand break repair. In a dual-flank Balb/cJ allograft model, local intratumoral administration of CFZ followed by ionizing radiation (8 Gy × 2) markedly suppressed primary tumor growth compared with monotherapies without inducing systemic toxicity. Although a strong abscopal effect on distant tumors was not observed, the combination treatment reduced the pulmonary metastatic burden relative to controls. Collectively, these results demonstrate that liposomal carfilzomib can act as an effective radiosensitizer, functioning through perturbation of DNA repair and modulation of the tumor response to radiation. This study highlights a translationally relevant nanotherapeutic approach for enhancing chemoradiotherapy outcomes in solid malignancies.

## Graphical abstract

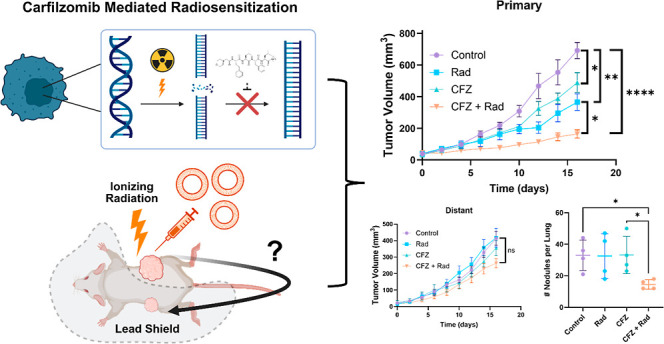

## 1 Introduction

It is estimated that over 2 million new cancer diagnoses will be made over 2025.^1^ Modern cancer treatments encompass chemotherapy, targeted therapy, radiotherapy, and surgical resection, among others.^2–5^ Radiation therapy is used in nearly 50% of all cancer treatments.^6^ However, as a monotherapy, radiotherapy is rarely curative.^3^ As a result, radiation therapy is often used in combination with other treatment modalities such as chemotherapy and immunotherapy.^3,7^ As such, there is a constant need to investigate potential combinations with strong synergy for more effective treatments.

Carfilzomib (CFZ) is a tetrapeptide epoxyketone covalent proteasome inhibitor that is currently FDA-approved for multiple myeloma.^8^ CFZ irreversibly binds and inhibits the β5 subunit of the 20S proteasome, a key component of the ubiquitin proteasome system (UPS).^9^ Proteasomal inhibition can influence a litany of cellular processes including cell cycle, DNA repair, apoptotic regulation, and a variety of tumorigenic pathways.^9–13^ For example, coadministration of a proteasome inhibitor and a topoisomerase inhibitor greatly increases the number of double-strand DNA breaks vs topoisomerase alone.^14^ DNA double-strand breaks are the primary driver in radiotherapy-mediated cell death.^15–17^

Intriguingly, CFZ has been investigated as a potential modulator of the immunosuppressive tumor microenvironment. Zhou et al. screened various compounds for the ability to reprogram protumor M2 macrophages to a proinflammatory and antitumor M1 macrophage phenotype.^18^ They found that proteasome inhibitors, including carfilzomib, promoted the secretion of proinflammatory cytokines in bone marrow-derived macrophages despite the presence of M2-stimulating IL-4. In mice, they demonstrated that carfilzomib treatment encouraged predominately the M1 phenotype over the M2 phenotype of tumor-associated macrophages (TAMs). Furthermore, the modulation of the TAMs was associated with increased infiltration of T cells into the tumors. Localized radiotherapy can induce shrinking of the distant metastasis in a process known as the abscopal effect. It is believed that the abscopal effect is driven by the generation of an immune response at the site of local treatment.^19^ M2 macrophages will limit generation of systemic antitumor immunity needed for an abscopal response in radiotherapy.^20^

We hypothesize that carfilzomib will improve radiotherapy of solid tumors through perturbation of DNA repair as well as through systemic immune activation and shrinking of distant tumors (abscopal effect). To test the potential immunotherapy, we used a dual tumor abscopal model on immunocompetent Balb/cJ mice.^20,21^ Lastly, carfilzomib was intratumorally injected to better control for true abscopal effects on the distant tumor. One of the key issues with carfilzomib is its limited water solubility. For the intratumoral injection, we used liposome vehicles because of their high biocompatibility and ability to solubilize hydrophobic drugs such as CFZ.^22^ Clinically, CFZ is administered with cyclodextrin.^23^ However, we want to avoid potential known immunostimulatory effects of cyclodextrin excipients and perform administrations using liposome vehicles.^24,25^

## 2 Materials and Methods

### 2.1 Materials

All cell culture reagents were purchased from ATCC and ThermoFisher unless otherwise stated. Lipids were procured from Avanti and NOF North America. CFZ was purchased from MedChemExpress.

### 2.2 Cell Culture

4T1 cells, procured from ATCC, were cultured in RPMI1640 media supplemented with 10% fetal bovine serum (FBS) and 1% penicillin/streptomycin at 37 °C in an atmosphere of 5% CO2. Cells were tested for murine pathogens prior to the animal studies.

### 2.3 Clonogenic Assay

Cells were seeded in 6-well plates at densities corresponding to the severity of treatment, ranging from 1000 cells per well up to 16,000 cells per well. Cells are allowed to adhere overnight. CFZ was added to 15 nM, and cells were incubated for 6 h prior to irradiation. Ionizing radiation was delivered via the Precision X-ray X-Rad 320 cabinet X-ray system at doses of 2, 4, 6, or 8 Gy. Cells were incubated for another 18 h in media containing CFZ prior to an exchange with fresh media. Colony formation then occurred over the next 7 to 10 days. Colonies were washed with 1xPBS and then fixed and stained with a 6% glutaraldehyde, 0.5% crystal violet solution for 30 min. Colonies were washed with tap water and then counted manually. Each colony contained at least 50 cells. Survival curves were fit to a nonlinear quadratic equation (above) by using GraphPad Prism software. Plots were normalized to the 0Gy treatment group to control for the cytotoxicity of CFZ itself.

$$\%Survival=100e^{-\left(\alpha x+\beta x^{2}\right)}$$

### 2.4 γ-H2AX Foci Retention

Cells were seeded in 8-well chamber slides at a density of 5 × 10^4^ cells per well and then allowed to adhere overnight. CFZ was added to 15 nM, and cells were incubated for 6 h prior to irradiation. Ionization radiation was delivered at a dose of 5 Gy. Cells were then incubated for another 18 h prior to fixing and staining. Cells were washed with 1xPBS and then fixed with 4% paraformaldehyde in 1xPBS for 30 min at room temperature. Cells were then washed with tris-buffered saline (TBS) and permeabilized with 0.25% Triton-X in blocking buffer (1x Blocker BSA in TBS, ThermoFisher) for 10 min at room temperature. Cells were then incubated in blocking buffer for 1 h at room temperature. After blocking, cells were incubated with rabbit anti-γ-H2AX antibody (Abcam, 1:500 dilution in blocking buffer) overnight at 4̊C. Cells were then washed twice with wash buffer (0.05% Tween-20 in TBS) for 5 min each at room temperature. Then, cells were incubated with fluorescein isothiocyanate (FITC)-conjugated goat antirabbit secondary antibody (Abcam, 1:500 dilution in blocking buffer) for 1 h at room temperature. Lastly, slides were washed with TBS prior to mounting in Fluoroshield with 4′,6-diamidino-2-phenylindole (DAPI) (Millipore Sigma) mounting media. Slides were analyzed using a Zeiss LSM880 confocal laser scanning microscope. γ-H2AX foci were identified (pixel clusters greater than 9) and manually counted on a per-cell basis. The number of foci/cells was determined from at least 100 cells for each treatment group.

### 2.5 Liposome Synthesis

Liposomal carfilzomib was synthesized via the thin film hydration method.^26^ Lipids and drugs were dissolved in chloroform in a round-bottom flask. Liposome composition was at a molar ratio of DOPC/cholesterol/DSPE-mPEG2K/CFZ of 62:30:5:3 (PEG-LP). The organic solvent was removed via a vacuum to form a thin film. The film is hydrated with 1xPBS for 60 min at room temperature. The crude liposome suspension was subjected to sonication via a Qsonica probe sonicator. The sonication treatment was 5 min at intervals of 2s on and 2s off at an amplitude of 26% while submerged in an ice bath (∼0 °C). After sonication, the liposome suspension was passed through a 0.45 μm polytetrafluoroethylene syringe filter to remove insoluble CFZ and large, aggregated particles.

### 2.6 Liposome Characterization

The encapsulation efficiency of liposomes was determined via HPLC. A 10 μL portion of filtered liposome suspension was diluted with 190 μL of methanol and then subjected to a 5 min bath sonication at room temperature. Solutions were then centrifuged at 10K RPM for 30 min and then filtered with 0.22 μm Teflon syringe filters prior to HPLC analysis. Liposomal drug encapsulation was quantified using HPLC with an Agilent XDB-C18 column. The chromatographic separation employed a mobile phase composed of water and acetonitrile containing 0.1% formic acid. An isocratic elution program (55:45 ACN/H_2_O) was run at a flow rate of 0.5 mL/min, resulting in a CFZ retention time of approximately 3.6 min. Quantification was performed by monitoring the absorbance at 210 nm, and drug concentrations were calculated by comparing integrated peak areas against a standard curve generated from serial CFZ dilutions. Particle size and zeta potential were determined using a Malvern Zeta Sizer Nano S equipped with a 632 nm laser. Liposome suspensions were diluted to 0.4 mg/mL for analysis. For cryogenic transmission electrons, samples were vitrified using a Vitrobot Mark IV system (Thermo Scientific) on Quantifoil R 2/2 copper grids (200 mesh; Quantifoil, Germany). Prior to sample application, grids were glow discharged at 15 mA for 30 s by using a PELCO easiGlow system. For each grid, 3.5 μL of the nanoparticle suspension was deposited under controlled conditions of 4 °C and 100% relative humidity, followed by blotting for 4 s. Cryo-electron microscopy data were collected on a Titan Krios G3 microscope (Thermo Scientific) equipped with a Falcon 4 direct electron detector. Imaging was performed at an accelerating voltage of 300 keV, using a nominal magnification of 37,000×, a spot size of 6, and a C2 aperture, yielding a calibrated pixel size of 1.8 Å. Drug release kinetics were determined via a dialysis experiment. Release of CFZ from a PEG LP nanoparticle was compared to CFZ in the clinically used cyclodextrin formulation (10 mM sodium citrate, 50:1 sulfobutylether beta-cyclodextrin:CFZ by mass, pH 3.5). CFZ formulations were placed in Slide-A-Lyzer 1 mL dialysis devices (Thermo Fischer) and dialyzed against 1xPBS at 37 °C. Samples were taken from the dialysis device at various time intervals, and CFZ content was quantified using the HPLC protocol above.

### 2.7 Liposome In Vitro Efficacy

A 3-(4,5-di methyl thiazol-2-yl)-2,5-diphenyltetrazolium bromide (MTT) assay was used to assess in vitro cytotoxicity of liposomal CFZ formulations. 4T1 cells were seeded at 4000 or 2000 cells/well for 48 h and 72 h experiments, respectively. Cells were allowed to adhere overnight. CFZ in liposomal and free form was added to the wells at concentrations ranging from 400 nM to 0.781 nM. Cells were incubated with CFZ for 48 or 72 h. Then, MTT reagent was added to 0.5 mg/mL. Cells were incubated with MTT reagent for 3 h prior to removal of media and solubilization of crystals with dimethyl sulfoxide. The absorbance of the wells was measured at 570 nm. Cell viability was determined via the equation below.

$$\%\;Cell\;Viability=100^{\times }\frac{{Abs}_{sample}-{Abs}_{blank}}{{Abs}_{control}-{Abs}_{blank}}$$

CFZ-mediated proteasome inhibition was accessed using the Amplite Fluorometric Proteasome 20S Activity Assay kit. 4T1 cells were seeded in 6-well plates at a density of 5 × 10^5^ cells per well. Cells were allowed to adhere overnight. CFZ in free or liposomal form was added to the wells to 10 nM. Cells were then incubated for 6 h. Cells were then washed with 1xPBS, lysed, sonicated, and centrifuged at 14000 rpm for 10 min. The supernatant was analyzed for both proteasome activity and protein content. The proteasome assay required 25 μL of lysate supernatant to be mixed with 75 μL of LLVY-R110 working buffer from the kit. The mixture was incubated at 37 °C for 60 min, and then fluorescence was measured at an excitation of 490 nm and emission of 525 nm. Fluorescence values were normalized to protein content determined by a bicinchoninic acid assay. All values were normalized to untreated control proteasome activity.

### 2.8 Mice

Balb/cJ mice were purchased at 5 weeks of age from The Jackson Laboratory. Animals were maintained in the Animal Veterinary and Biomedical Sciences Building at Pennsylvania State University. All animal protocols were approved by the Pennsylvania State University Institutional Animal Care and Use Committee (IACUC, PROTO202402651)

### 2.9 In Vivo Tumor Growth Experiments

The dual tumor abscopal model utilized Balb/cJ mice inoculated with 2 tumors, one on each flank. Mice were shaved prior to tumor injection. A 4T1 cell suspension at 10^7^ cells/mL was mixed with equal volumes of Corning Matrigel basement membrane. A total of 10^6^ cells (200 μL) was injected into the right flank, forming the “primary” tumor, while 2.5 × 10^5^ cells (50 μL) were injected into the left flank, forming the “distant” tumor. Tumors developed over the course of 5 days prior to initial treatment. The following 4 treatment groups were used (*n* = 4 or 5): (1) control, (2) radiation only (Rad), (3) CFZ only, and (4) CFZ and radiation (CFZ + Rad). On the first day of treatment, mice were intratumorally injected with 1 mg/kg CFZ at a volume of 30 μL. Six hours later, mice received their first dose of radiation in the form of an 8Gy fraction delivered via the X-rad 320 cabinet X-ray system. Mice were protected with a lead shield that only exposed the “primary” tumor while protecting the rest of the mouse, specifically the “distant” tumor on the left flank. A week later, mice received the same treatment with another 1 mg/kg dose of CFZ and an 8Gy fraction of radiotherapy. Superficial flank tumors were measured with digital calipers for length (*L*) and width (*W*). Total tumor volume was determined by the following formula: tumor volume = 0.5*L*W*W. Mice were monitored for 16 days after the first treatment began prior to euthanasia, tissue harvest, and hematoxylin and eosin (H&E) staining. Area under curve (AUC) analysis of tumor growth curves was done through GraphPad Prism software.

### 2.10 Statistical Analysis

Data was plotted and analyzed using GraphPad Prism software. Statistical significance was determined using a two-tailed *t*-test when comparing 2 groups or a one-way ANOVA with Tukey correction when more than 2 groups are being compared at once unless otherwise noted. An n = 3 was used in all experiments unless otherwise stated. Plots are displayed as the mean ± the SD unless otherwise stated.

## 3 Results

### 3.1 Radiosensitization and γ-H2AX Foci Retention

A clonogenic assay was performed to determine the radiosensitization capabilities of CFZ. 4T1 cells were incubated with 15 nM CFZ for 6 h prior to X-ray irradiation. Eighteen hours after irradiation, the media was exchanged, and colonies were allowed to form. A decrease in survival post-irradiation for CFZ-treated cells vs untreated cells was observed (Figure 1A). The dose enhancement at 37% survival was 1.26. This value was calculated after fitting the survival curves to a linear quadratic model (Table 1).

**Figure 1:**
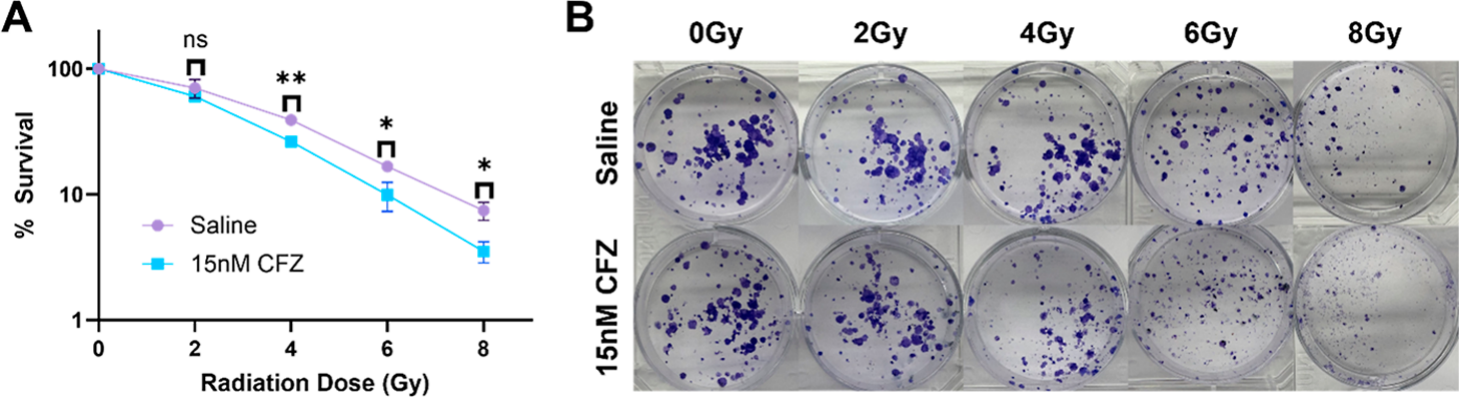
CFZ sensitizes 4T1 cells to radiation: (A) survival curve derived from the clonogenic assay for cells treated with saline vs 15 nM CFZ. Survival at each radiation dose was compared via the two-tailed *t*-test (ns = nonsignificant, *p < 0.05, **p < 0.01). (B) Images of plates representative of survival for each treatment.

**Table 1:** Results from Nonlinear Regression of Clonogenic Survival Data to a Linear Quadratic Equation

best-fit values	saline	CFZ
Α	0.1203	0.1903
Β	0.02839	0.03363
α/β	4.238	5.659
95% CI (profile likelihood)		
Α	0.05514 to 0.1841	0.1498 to 0.2298
Β	0.01421 to 0.04519	0.02311 to 0.04552

To detect the presence of double-strand DNA breaks, a γ-H2AX immunofluorescence assay was performed. Cells were incubated with CFZ for 6 h, followed by a 5Gy dose of radiation. Cells were fixed and stained 18 h after radiation. Cells treated with both CFZ and radiation had a significantly higher number of γ-H2AX foci compared to radiation alone (****p < 0.0001, Figure 2A). CFZ alone did not have significantly more γ-H2AX foci than the untreated control (ns, *p* > 0,05).

**Figure 2:**
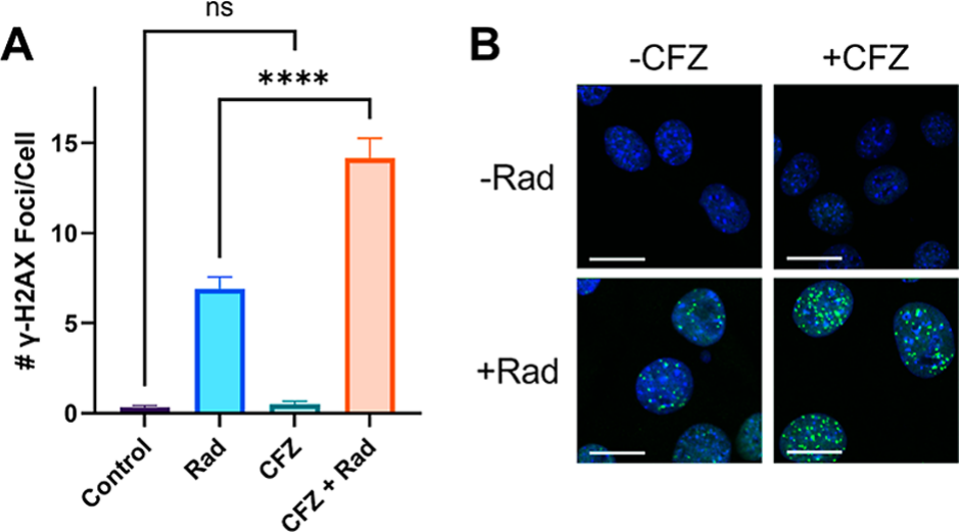
CFZ perturbs DNA repair in response to radiation: (A) γ-H2AX foci retention assay plotted as the number of foci/cell (mean ± SEM). Values were compared via one-way ANOVA (ns = nonsignificant, ****p < 0.0001). (B) Representative images of 4T1 cells stained for γ-H2AX after various treatments. Blue represents DAPI-stained nuclei, and green represents γH2AX foci. Scale bar: 10 μm.

### 3.2 CFZ-Loaded Liposome Characterization and In Vitro Efficacy

CFZ liposomes were synthesized via thin film hydration and probe sonication for size reduction. Liposomes had a mean z-average hydrodynamic diameter of 127.4 nm ± 0.5 (SD) and a zeta potential of −15.6 ± 0.7 (SD). Size distribution plots for liposomes with and without CFZ are shown as well as Cryo-TEM micrographs (Figure 3A–C). Mean encapsulation efficiency was found to be 63.9 ± 2.5%. Drug release kinetics were investigated with a dialysis experiment. Cyclodextrin formulation of CFZ demonstrated a burst of 64% of the drug in 2 h vs the PEG-LP formulation with only 14% (Figure S1). Cytotoxicity of CFZ-loaded liposomes was investigated with an MTT assay on 4T1 cells treated with various doses of CFZ. Dose–response curves comparing 48 and 72 h treatments with free, solution-phase CFZ or CFZ encapsulation within a liposome were determined. Liposomal CFZ was found to be more potent than CFZ for 48 h for several CFZ concentrations with similar potency observed over 72 h (Figure 3D,E). To ensure bioactivity of CFZ, a proteasome activity assay was performed. Cells treated with free solution phase CFZ, liposomal CFZ, or a control were lysed. The lysate was assayed for proteasome activity. The cell treated with PEG liposome CFZ saw a decrease in proteasome activity when compared to free CFZ (*p* < 0.05; Figure 3F).

**Figure 3:**
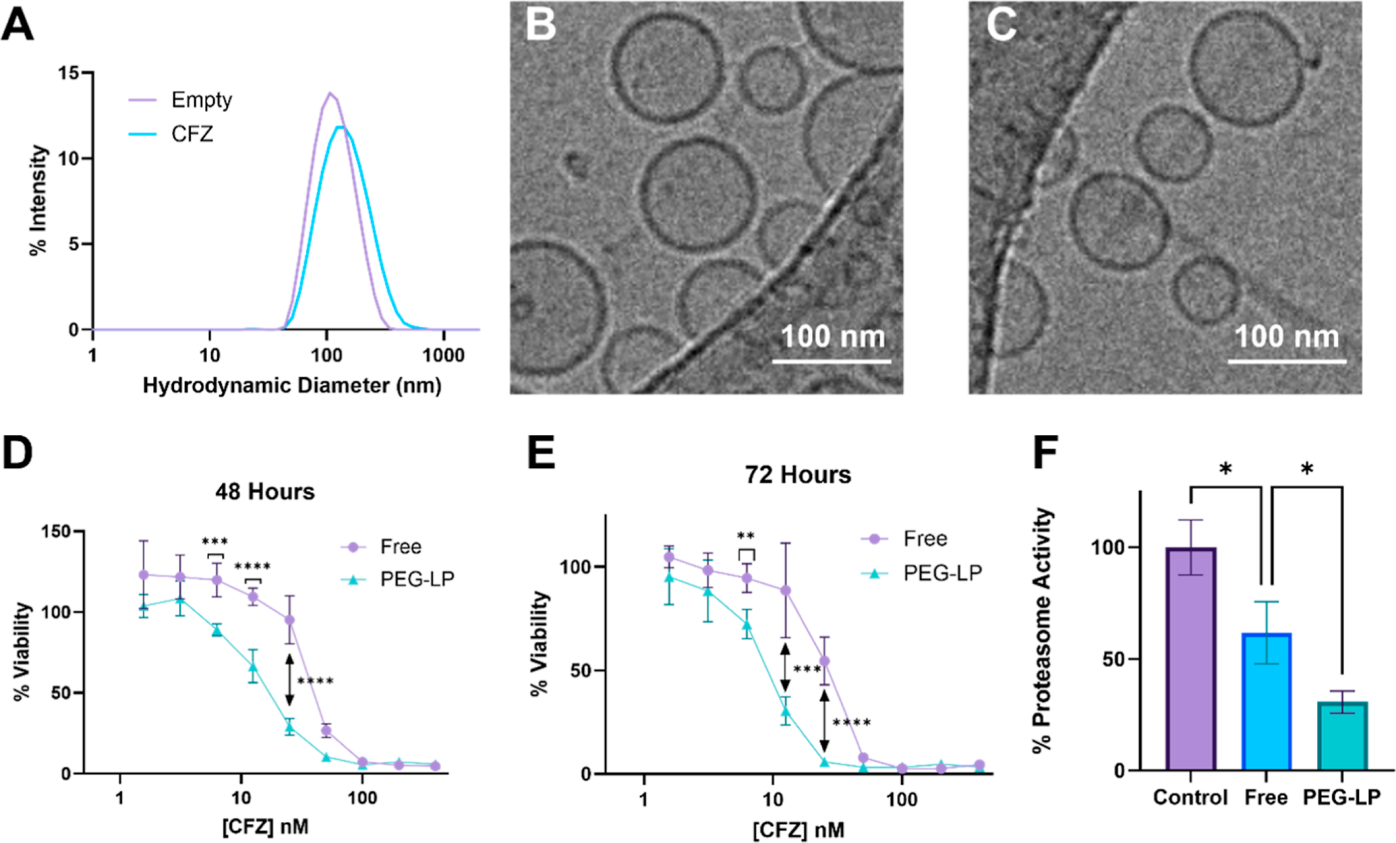
Liposomal CFZ was successfully synthesized and exhibited efficacy in vitro. (A) Dynamic light scattering distribution curves for liposomes loaded with CFZ and empty controls. (B) Cryo-TEM micrograph for empty control liposomes. (C) Cryo-TEM micrograph for CFZ-loaded liposomes. (D) 48 h dose–response curve. % Viability at various CFZ concentrations compared via the two-tailed *t*-test (***p < 0.001, ****p < 0.0001). (E) 72 h dose–response curve. % viability at various CFZ concentrations compared via the two-tailed *t*-test. (F) Proteasome activity assay; values were compared via one-way ANOVA (*p* < 0.05).

### 3.3 In Vivo: Primary Tumor

A dual flank tumor model was applied to investigate the potential abscopal effect. Mice were grafted with a tumor in the right flank dubbed “primary” as well as the left flank dubbed “distant” (Figure 4A). The right flank was directly treated via intratumoral injection of CFZ. During irradiation, the mouse was covered with a lead shield that exposed only the right flank to ionizing radiation. Therefore, the left flank was never treated directly to control for exogenous treatment that triggered tumor regression. Figure 4B depicts the treatment protocol for dual flank tumor mice. Mice treated with both CFZ and radiation exhibited decreased tumor growth in the primary tumor versus control (*p* < 0.0001), CFZ alone (*p* < 0.01), and radiation alone (*p* < 0.05) (Figure 4C). Area under curve (AUC) analysis demonstrated similar results with CFZ and radiation treatment having lower AUC than control (*p* < 0.0001), CFZ alone (*p* < 0.01), and radiation alone (*p* < 0.05) (Figure 4D).

**Figure 4:**
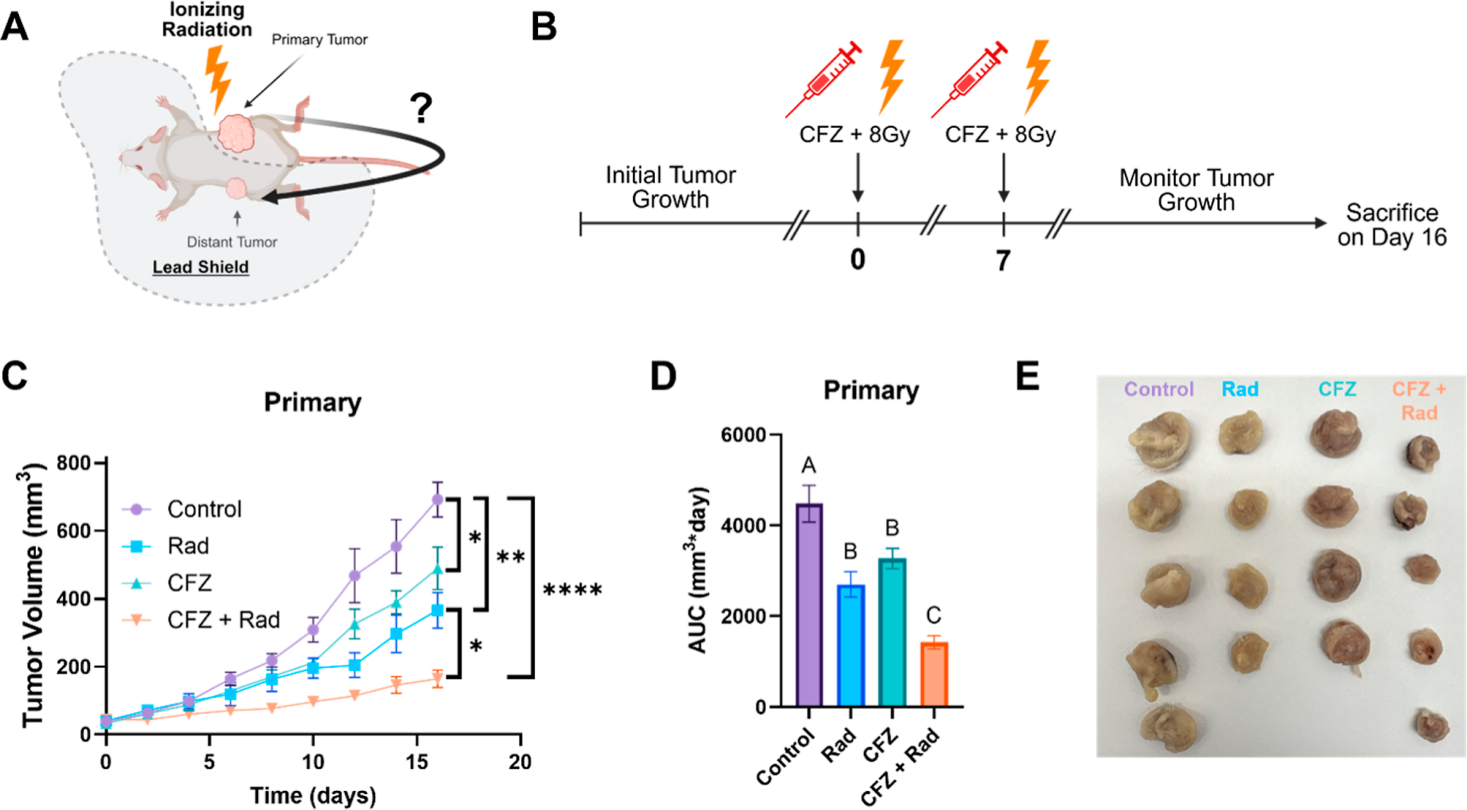
Combination of CFZ and radiotherapy suppresses primary tumor growth: (A) schematic treatment protocol. Mice with two tumors will have localized therapy on a single tumor, while the other tumor serves to detect a potential abscopal effect. (B) Treatment schedule: mice will be treated twice with intratumoral injection of CFZ (1 mg/kg) followed by irradiation with 8Gy. Treatments are separated by 7 days. (C) Tumor growth curve for the primary tumor (*n* = 4 or 5 plotted as the mean ± SEM). Final day volumes were compared via one-way ANOVA (*p < 0.05, **p < 0.01, ****p < 0.0001). (D) AUC analysis performed on curves from panel (C). Total AUC in mm^3^*day was plotted, and values were compared via one-way ANOVA. Comparisons are shown in a compact letter display to demonstrate significantly different groups (*p* < 0.05). (E) Image of primary tumors dissected from animals after completion of the study.

### 3.4 In Vivo: Distant Tumor and Lung Metastasis

In contrast to the primary tumor, there was little treatment efficacy observed with the distant tumor. CFZ and radiation did not reduce tumor growth significantly compared to other treatment groups (*p* > 0.05). AUC analysis showed that CFZ + radiation reduced distant tumor burden compared to the radiation alone group (*p* < 0.05) but was not significantly different from control and CFZ alone (*p* > 0.05). The effects of CFZ and radiation on the formation of new metastases were examined by quantifying metastatic nodules on the lungs (Figure 5C). The combination treatment of CFZ and radiation decreases the number of new metastases when compared to the control and CFZ alone. However, we did not observe significant differences between the number of metastases in radiation-only-treatment mice and CFZ- and radiation-treated mice.

**Figure 5:**
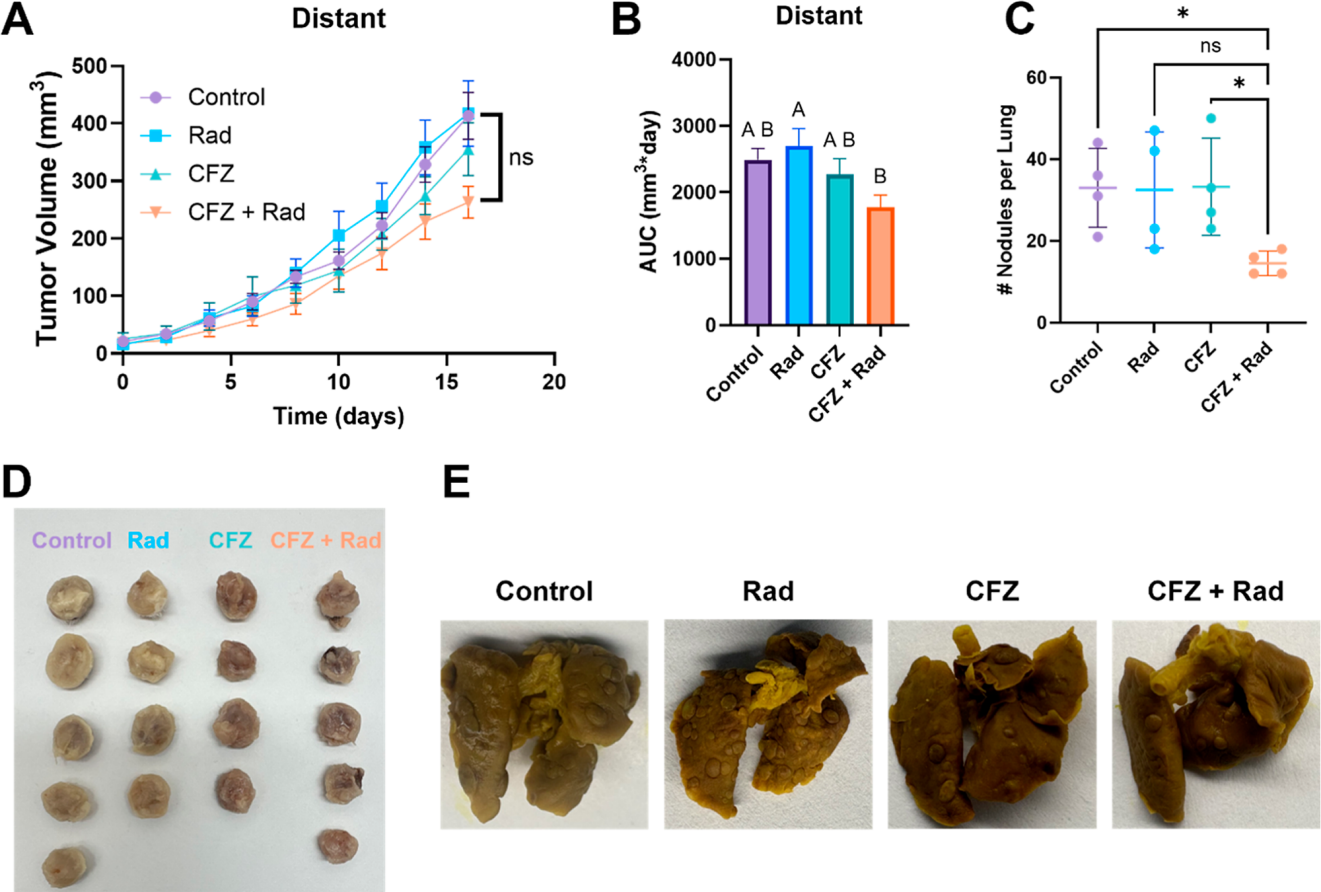
Local therapy does not slow the growth of the established distant tumor but does hamper the formation of lung metastasis: (A) tumor growth curve for the distant tumor (*n* = 4 or 5 plotted as the mean ± SEM). Final day volumes were compared via one-way ANOVA. No statistically significant differences were observed (ns = nonsignificant, *p* > 0.05). (B) AUC analysis was performed on curves from panel (A). Total AUC in mm^3^*day was plotted, and values were compared via one-way ANOVA. Comparisons are shown in a compact letter display to demonstrate significantly different groups (*p* < 0.05). CFZ + Rad was significantly different from the Rad-only group (*p* < 0.05) but was not different from the control group (*p* > 0.05). (C) Number of metastatic nodules formed on lungs dissected from mice undergoing various treatments (*n* = 4). Values were compared using a Brown-Forsythe and Welch ANOVA (ns = nonsignificant with *p* > 0.05 and *p < 0.05). D) Images of distant tumors dissected from animals after completion of the study. (D) images of lungs dissected and stained with Bouin’s stain for visualization.

### 3.5 In Vivo: Treatment Tolerability

The treatments used in this study were well tolerated. Mouse weights were measured throughout the study with no significant differences observed (Figure 6A). Mice treated with radiation had significantly lighter spleens when compared to mice that did not receive radiation (*p* < 0.05, Figure 6B). However, there were no differences observed in terms of spleen weight when comparing mice treated with CFZ and without CFZ (*p* > 0.05). Organs were harvested and stained with H&E stain. Images of the stained organs can be seen in Figure 6C. There are no major changes to the histological morphology of any organs compared to the control.

**Figure 6:**
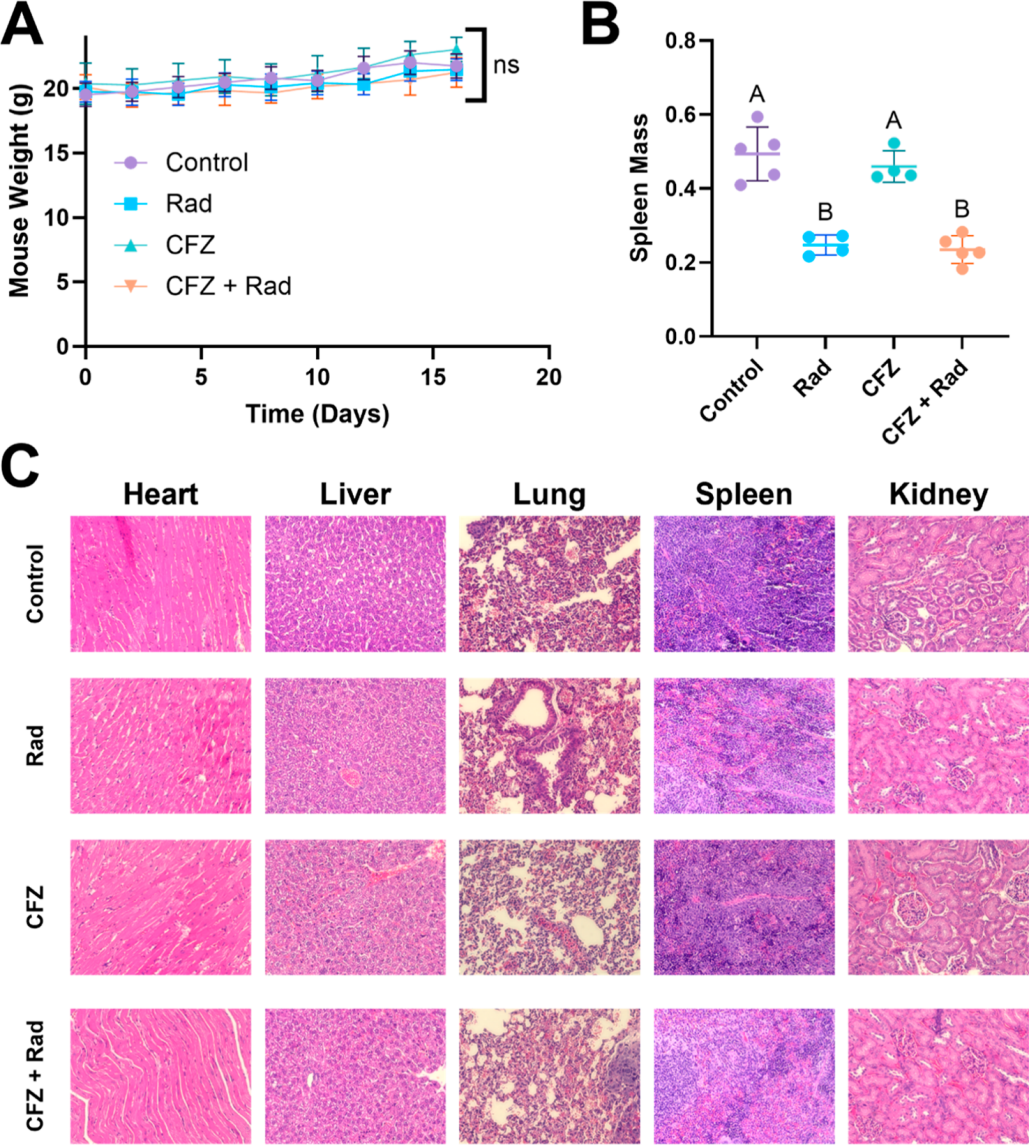
CFZ chemoradiotherapy was well tolerated: (A) Mouse weights plotted over the course of the study. Day 16 weights were compared using one-way ANOVA (ns, *p* > 0.05). (B) Spleen weights compared using one-way ANOVA with statistical comparison displayed using a compact letter display (*p* < 0.05). (C) H&E-stained tissue slices from organs harvested after completion of the treatments.

## 4 Discussion

Chemoradiotherapy is a critical tool in cancer treatment. Typical radiosensitizing chemotherapeutic drugs include anthracyclines, topoisomerase inhibitors, antimetabolites, platinum drugs, and alkylating agents.^6^ A key commonality among these drugs is a direct interaction with DNA. Intuitively, drugs that damage or alter DNA will synergize with DSB-inducing ionizing radiation. However, complex cellular systems allow for a plethora of targets for radiosensitization. One such target is the proteasome. The UPS is key in the cellular recycling of damaged and misfolded proteins, but it also plays a key role in the regulation of many cellular activities.^10^ Critically, one such activity is DNA repair.^12,14,27–29^ To the best of our knowledge, CFZ has yet to be studied as a radiosensitizer.

In this study, we were able to provide preliminary evidence that CFZ can behave as a radiosensitizer. The clonogenic assay is the gold standard for determining radiosensitivity for in vitro models.^30^ 4T1 cells treated with CFZ for 6 h prior to radiation and for 18 h post irradiation saw decreased clonogenic survival when compared to control (Figure 1A). This critical finding then prompted additional studies to determine what may be the driver of the radiosensitization. In the response to double-strand breaks, cells can form γ-H2AX foci.^31^ We demonstrated that the coadministration of CFZ and radiation increased the mean number of γ-H2AX foci per cell (Figure 2A). Additionally, CFZ alone did not produce a significantly higher number of foci versus the untreated control. As a result, we conclude that CFZ perturbs the repair pathway of double-stranded breaks. This finding supports the radiosensitization activity observed in the clonogenic assay.

CFZ is marred by poor water solubility. Clinically, CFZ is administered in a solution with a high molar excess of cyclodextrin. High doses of cyclodextrin have been associated with nephrotoxicity.^32^ As a result, we have decided to use liposomes as a delivery vehicle. Liposomes are well tolerated and work well to encapsulate hydrophobic drugs, thereby boosting the solubility.^33^ We successfully encapsulated CFZ in liposomes with an encapsulation efficiency of 63.9%. In vitro efficacy was validated via a dose–response assay and proteasome inhibition assay. In both cases, liposomal CFZ was shown to be more potent, with greater effects for the same concentration. The liposomal formulation may better facilitate CFZ uptake into cells or improve stability prior to cellular uptake.^34^

The dual flank tumor 4T1 allograft provides a robust model for investigating radiosensitization, as well as potential immunotherapy. Analysis of the primary tumor will give insight into radiosensitization capabilities of CFZ. It was observed that mice treated with both CFZ and radiotherapy had less tumor growth compared to CFZ or radiation alone. This finding supports the in vitro clonogenic assay showing decreased survival of cells treated with both CFZ and radiation. However, the magnitude of the therapeutic improvement with combination therapy is small. This may be due to a myriad of factors including insufficient uptake of nanoparticles or a suboptimal radiotherapy fractionation schedule. Additionally, despite intratumoral administration, heterogeneous intratumoral distribution and limited penetration into hypoxic or poorly perfused tumor regions may restrict effective drug–radiation synergy.^35^ Such spatial and microenvironmental constraints can impose an upper limit on the therapeutic benefit achievable with local combination therapy.^36–38^

While the primary tumor can be used to study cellular-level radiosensitization in an animal model, the distant tumor allows us to investigate potential immunotherapy. The abscopal effect occurs when localized tumor therapy facilitates the shrinking of distant metastasis.^19^ In the context of this study, the primary tumor was treated locally with both CFZ and radiation. The left flank “distant” tumor served as a marker for abscopal effects. The abscopal effect is rare clinically and does not represent a reliable treatment modality.^39^ The precise mechanism is under debate; however, one plausible explanation is radiation mediates the activation of the immune system.^40^ Once ionizing radiation begins killing cancer cells, damage-associated molecular patterns (DAMPs) are released, thereby triggering antigen-presenting cells to mature and ultimately migrate to lymph nodes, where they can support generation of activated T cells targeted toward cancer-specific antigens.^41^ As previously mentioned, the abscopal effect is rare, and in practice, the proposed mechanism rarely occurs. This is due to the immunosuppressive microenvironment in cancers including immunosuppressive cytokines, protective receptors, and M2 macrophages.^45^ CFZ’s multitude of cellular effects extends to macrophages with apparent polarization to M1, antitumor, phenotype.^18^ The immunomodulatory nature of CFZ inspired the hypothesis that CFZ administration may produce the necessary environment for the abscopal effect in response to radiotherapy. However, based on the results presented in Figure 4F, there was no significant abscopal effect observed. As mentioned above, many factors play a role in an immunosuppressive tumor microenvironment. While CFZ may polarize macrophages to the favorable M1 phenotype, other factors such as overexpression of PD-1 may help tumor cells avoid the immune system.^42^ Another factor in the production of an abscopal response is the radiotherapy treatment protocol. Yin et al. investigated the combination of TLR7/8 agonist prodrugs with radiotherapy as a potential abscopal-inducing therapy.^43^ They observed that fractionation strategies can influence the shrinking of distant tumors. For example, a single 20Gy fraction produces no shrinking of distant tumors compared to three 6Gy fractions (18Gy total), which induces significant growth control in the distant tumor. An additional factor influencing the absence of a significant abscopal response is the inherently aggressive and highly immunosuppressive nature of the 4T1 allografted tumor model, which is well-known to resist systemic antitumor immune activation. This shortcoming of the current study more accurately reflects the practical issues posed by immunologically treatment-resistant malignancies. In this setting, our radiation fractionation schedule (8 Gy × 2), although sufficient for local tumor management, may be inadequate for effective immune priming. Recent results showing improved abscopal responses using various fractionation procedures when combined with immunomodulatory drugs, such as TLR agonists, underscore a significant avenue for further research beyond the present study.^43^

Despite the lack of efficacy in suppressing distant tumor growth, a decrease in the number of lung metastases formed was observed for CFZ + radiation treatment groups versus control and CFZ-only groups. However, a statistically significant difference between the formation of lung metastasis in the radiation-only groups and in the CFZ + radiation group was not observed. This finding may suggest that CFZ in combination with radiation can prime the immune system to stunt metastatic tumors from forming but is not powerful enough to affect established tumors. This phenomenon will require further investigation.

In the present study, we demonstrated the utility of CFZ as a radiosensitizer in a 4T1 allograft solid tumor model at both the in vitro and the in vivo levels. This prompts studies on human cell lines and xenograft models to better understand and predict potential applications in the clinic. Despite the lack of observed abscopal effect, CFZ has been studied in combination with other treatments including antibodies against PD1, PDL1, and CTLA-4; immunostimulatory agents such as IL-2; and immunomodulatory small-molecule drugs.^44^ Furthermore, alternative radiotherapy treatment schedules can be tested to identify potential doses and fractionation patterns that may better support generation of systemic antitumor immunity.^45^

## 5 Conclusions

This study demonstrates that liposome-encapsulated carfilzomib (CFZ) functions as an effective radiosensitizer in a solid tumor model, offering a promising strategy to enhance the therapeutic efficacy of chemoradiotherapy. Through in vitro and in vivo analyses, we show that CFZ amplifies radiation-induced DNA double-strand break retention and decreases clonogenic survival in 4T1 carcinoma cells, consistent with proteasome inhibition–mediated disruption of DNA repair pathways. Liposomal formulation markedly improved CFZ solubility, bioactivity, and tolerability, enabling localized intratumoral administration without systemic toxicity. In vivo, the combination of CFZ and ionizing radiation significantly suppressed primary tumor growth and reduced pulmonary metastatic burden, although a robust abscopal effect on distant tumors was not observed. Together, these findings identify proteasome inhibition via liposomal CFZ as a viable radiosensitization strategy and provide a foundation for further exploration of nanocarrier-enabled radioimmunotherapeutic combinations. Future work will focus on the mechanistic elucidation of CFZ-mediated DNA repair inhibition, optimization of radiotherapy dosing and fractionation, and combination with immune checkpoint blockade or immunostimulatory agents to potentiate systemic antitumor responses.
